# Place de la bili-IRM dans le diagnostic etiologique des icteres cholestatiques à Dakar

**DOI:** 10.11604/pamj.2016.24.174.9894

**Published:** 2016-06-29

**Authors:** Nfally Badji, Geraud Akpo, Hamidou Deme, Mouhamadou Hamine Toure, Mamadou Ly, Boucar Ndong, El Hadji Niang

**Affiliations:** 1 Service de Radiologie Générale du CHU Aristide Le Dantec, Dakar, Sénégal

**Keywords:** Bili-IRM, ictère cholestatique, étiologies, Bili- MRI, cholestatic jaundice, etiologies

## Abstract

La Bili-IRM est une méthode diagnostique d’introduction relativement récente dans l’arsenal d’exploration de la pathologie bilio-pancréatique. Il s’agit d’une technique fiable reproductible et non invasive, permettant la visualisation directe des voies biliaires et pancréatiques. Le but de ce travail était d’étudier les aspects morphologiques des principales anomalies et d’évaluer la place de la bili-IRM dans le diagnostic étiologique des ictères cholestatiques. Il s’agit d’une étude rétrospective réalisée sur 4 ans et six mois (Janvier 2008 à Juillet 2012) portant sur 17 patients colligés dans les services d’imagerie médicale du CHUN de Fann et de l’hôpital Principal de Dakar. Tous les patients ont bénéficié d’une IRM (1,5T) avec des protocoles adaptés en fonction de la pathologie explorée. Les dossiers retenus sont ceux dont le diagnostic a été établi à partir du bilan biologique et ayant bénéficié à la fois d’une bili-IRM et d’une exploration chirurgicale. Il s’agissait de 05 femmes et de 12 hommes soit un sexe ratio de 2.4. L’âge moyen des patients était de 58 ans avec des extrêmes de 35 et 81 ans. Les tumeurs de Klatskin étaient observées chez 07 patients avec une forme infiltrante dans 71% des cas et une forme exophytique dans 28% des cas. Les cancers de la vésicule biliaire étaient notés dans 28% des cas. Les cancers de la tête du pancréas représentaient 28% des cas. La lithiase de la voie biliaire principale était présente chez 05 patients avec un empierrement cholédocien dans 60% des cas et une lithiase unique dans 40% des cas. Toutes ces lésions étaient responsables d’une dilatation des VBIH. Un cas de dilatation des voies biliaires intra et extra hépatiques était retrouvé sans cause bilio-pancréatique. La bili-IRM est l’examen de choix dans l’exploration des ictères cholestatiques. Elle doit être demandée en première intention lorsqu’on suspecte une lithiase résiduelle et en deuxième intention après l’échographie lorsque cette dernière suspecte une obstruction tumorale des voies biliaires. Son association avec la TDM constitue la meilleure combinaison pour le diagnostic étiologique et le bilan préopératoire des obstructions biliaires tumorales.

## Abstract (en)

Biliary MRI is a relatively new diagnostic test in the arsenal of exploration techniques in biliopancreatic pathology. This is a reproducible and reliable non invasive technique for direct visualization of biliary and pancreatic ducts. This study aims to evaluate the morphological features of major abnormalities and the role of biliary MRI in the etiological diagnosis of cholestatic icteruses. This is a retrospective study of 17 patients conducted in the Imaging Unit of the University Hospital of Fann and of the Principal hospital of Dakar over a period of 4 years and six months (January 2008 at July 2012). All patients underwent MRI (1.5T) according to the standardized protocols for the explored pathology. Only medical records of patients whose diagnosis was established based on laboratory tests and who underwent biliary MRI and surgical exploration were retained. The study involved 5 women and 12 men with a sex ratio of 2.4. The average age of patients was 58 years, ranging between 35 and 81 years. Klatskin tumors were found in 7 patients with infiltrative form in 71% of cases and exophytic form was found in 28% of cases. Cancers of the gallbladder were found in 28% of cases. Cancers in the head of the pancreas accounted for 28% of cases. Major bile duct lithiasis was detected in 5 patients, choledocholithiasis in 60% of cases and a single lithiasis in 40% of cases. All these lesions were responsible for an expansion of intrahepatic bile duct (IHBD). One case of intra and extrahepatic bile ducts dilatation was found without biliopancreatic cause. Biliary MRI is the test of choice for the exploration of cholestatic icteruses. It should be recommended as first-line examination when residual lithiasis is suspected and as second-line examination after ultrasound, when the latter shows a suspected bile ducts tumoral obstruction. Its association with CT scan is the best combination of screening tests for etiologic diagnosis and pre-operative assessment of tumoral biliary obstructions.

## Introduction

La cholestase est l’ensemble des manifestations cliniques et biologiques liées à la diminution ou à l’arrêt de la sécrétion biliaire. Elle peut être secondaire soit à l’obstruction des voies biliaires soit à l’arrêt ou à la diminution de production de la bile par les hépatocytes [1]. L’ictère qui en découle est un signe d’alarme traduisant une pathologie des voies biliaires dont il faut rechercher la cause. L’examen clinique et les données biologiques permettront de différencier un ictère obstructif d’une maladie hépatique cholestatique. Devant le tableau clinique, l’imagerie est indispensable pour confirmer l’obstacle mécanique, établir le siège et poser le diagnostic étiologique. Actuellement le gold standard diagnostic dans la pathologie des voies biliaires est représenté par la cholangiographie par résonance magnétique ou bili-IRM qui permet une étude exhaustive de l’arbre bilio-pancréatique. Sa sensibilité et sa spécificité sont de loin supérieures à celles de la tomodensitométrie et de l’échographie. Dans ce travail, nous proposons de faire une étude rétrospective portant sur une série de bili-IRM. Le but de ce travail rétrospectif était d’étudier les aspects morphologiques des principales anomalies et d’évaluer la place de la bili-IRM dans le diagnostic étiologique des ictères cholestatiques.

## Méthodes

Il s’agissait d’une étude rétrospective réalisée sur 4 ans et six mois (Janvier 2008 à Juillet 2012) portant sur 17 patients colligés dans les services d’imagerie médicale du CHUN de Fann et de l’hôpital Principal de Dakar. L’âge moyen des patients était de 58 ans avec des extrêmes de 35 et 81 ans. Le sexe ratio était de 2.4. Tous les patients ont bénéficié d’une IRM (1,5T) avec des protocoles adaptés en fonction de la pathologie explorée. Les dossiers retenus sont ceux dont le diagnostic a été établi à partir du bilan biologique et ayant bénéficié à la fois d’une bili-IRM et d’une exploration chirurgicale. Nous avons étudié l’aspect des images d’arrêt (siège, morphologie, signal IRM), la nature de l’obstacle à l’écoulement de la bile (lithiase, tumeur, inflammation), les anomalies des parenchymes (hépatique, pancréatique) et les signes d’orientation thérapeutique.

## Résultats

### Résultats globaux

L’IRM avait permis de mettre en évidence une obstruction des voies biliaires chez 108 patients dont 17(15%) répondaient aux critères d’inclusion. Les trois examens (échographie, TDM et bili-IRM) étaient contributifs dans le diagnostic étiologique des ictères cholestatiques (Tableau 1).

**Tableau 1:** Resultats des différents examens

Etiologies	Echographie	TDM	IRM
Lithiase du cholédoque	03	05	05
Tumeur de klatskin	02	03	07
Cancer du pancréas	02	02	02
Cancer de la vésicule biliaire	02	02	02
Autres	00	00	01
**TOTAL**	**09**	**12**	**17**

### Aspects IRM: causes biliaires d’ictère cholestatique

#### Causes tumorales

**Figure 1:**
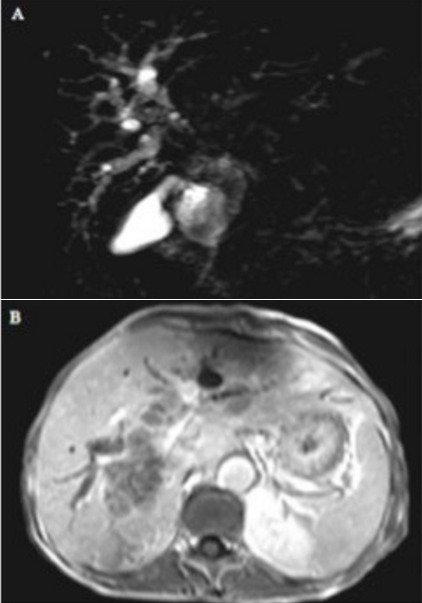
Tumeur de Klatskin: A) bili-IRM, séquence 2D montrant un arrêt abrupte de la convergence hilaire avec dilatation des voies biliaires intrahépatiques, cholédoque normal (type II klatskin); B) coupe axial T1 fatsat après injection de gadolinium, montrant une infiltration du hile hépatique avec dilatation des voies biliaires intrahépatiques (Hôpital Fann)

**Tumeurs de klatskin:** Six patients avaient présenté une tumeur de klatskin (Figure 1) type II de Bismuth et 01 cas de type IV. L’âge des patients était compris entre 41 et 70 ans avec une prédominance masculine. Ces tumeurs se présentaient à l’IRM sous forme de sténose ou d’amputation de la convergence hilaire et du canal hépatique commun pour le type II, en plus un défaut de convergence des canaux biliaires de premier ordre gauche et droit pour le type IV. Ces sténoses étaient responsables d’une dilatation en amont des voies biliaires intra hépatiques (VBIH). La forme infiltrante représentait 71% des cas et celle exophytique 28% des cas. Deux cas de métastases hépatique et vésiculaire étaient retrouvés.

**Figure 2:**
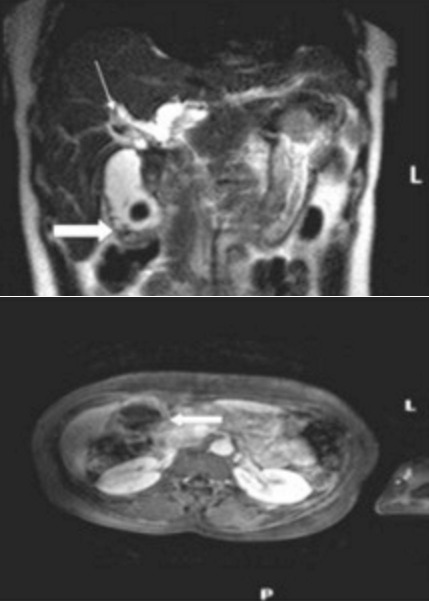
Tumeur de la vésicule biliaire: coupes coronale T2 et axiale T1 fatsat avec injection de gadolinium montrant un épaississement tumoral de l’infundibulum (grosse flèche) d’une vésicule biliaire lithiasique et une dilatation des VBIH (petite flèche), (Hôpital Fann)

**Cancer de la vésicule biliaire**: Le cancer de la vésicule biliaire (Figure 2) était retrouvé dans 02 (12%) cas avec un envahissement hépatique (segments IV et I) par contigüité. Ils réalisaient un épaississement pariétal focal de l’infundibulum, en hypo signal T1, en signal intermédiaire T2 et prenaient fortement le contraste.

**Figure 3:**
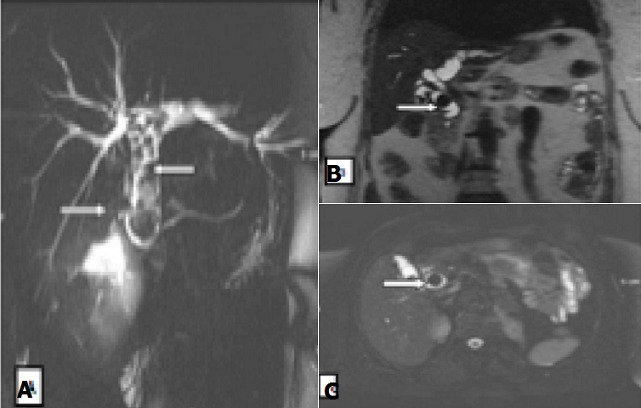
Lithiase de la voie biliaire principale: A) bili-IRM, séquence 2D montrant un empierrement cholédoque avec dilatation des voies biliaires intra hépatiques (Hôpital principal); B, C) coupes coronale et axiale en T2EG, montrant une lithiase enclavée dans le bas cholédoque (Hôpital Principal)

**Causes lithiasiques**: La lithiase du cholédoque (Figure 3) était observée chez 05 (29%) patients. Un empierrement cholédocien était retrouvé dans 60% des cas et une lithiase unique enclavée dans le bas cholédoque dans 40% des cas. Elles entrainaient toutes une dilatation des VBIH et extrahépatiques (VBEH) d’amont. Ces calculs se présentaient sous forme de structures vides de signal (hyposignal) T1 et T2, arrondies ou ovalaires toujours entourées d’un fin liséré en hyper signal T2 (labile).

**Figure 4:**
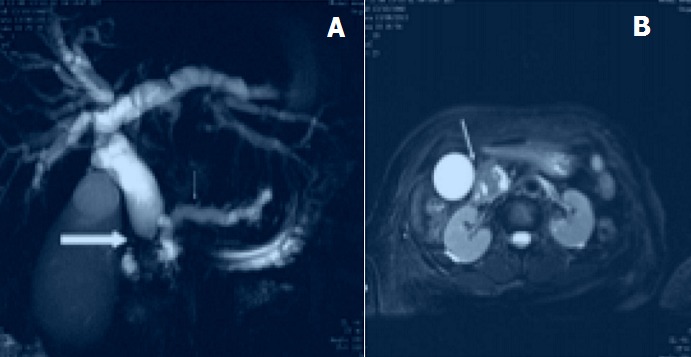
Tumeur de la tête du pancréas avec dilatation bicanalaire: A) Bili-IRM réalisée avec une séquence T2 RARE en projection de 40mm d’épaisseur dans un plan coronal, montrant une dilatation du cholédoque avec arrêt franc « en culot d’obus » (grande flèche) et du wirsung (petite flèche) en amont d’une tumeur pancréatique (Hôpital Principal); B) même patient, coupe axiale en séquence T2 fatsat, montrant la tumeur de la tête du pancréas (Hôpital principal)

**Causes pancréatiques**: Les cancers de la tête du pancréas (Figure 4) représentaient 12% des cas et étaient survenues sur des patients jeunes, de 41 et de 46 ans. Les lésions étaient en hypo signal hétérogène T1 et T2, et présentaient une discrète prise de contraste en T1 gadolinium. Elles étaient responsables d’une dilatation du wirsung et du cholédoque avec arrêt franc « en culot d’obus ». Pas d’envahissement vasculaire ni d’hypertrophie ganglionnaire décelés.

### Concordance entre les examens d’imagerie (Echographie-TDM)

Les résultats de l’échographie ont été comparés à ceux de la tomodensitométrie et une concordance a été retrouvée dans 77% des cas. Les résultats de l’échographie ont été comparés à ceux de la tomodensitométrie et une concordance a été retrouvée dans 77% des cas.

### Concordance entre bili-IRM et chirurgie

Dans notre série, tous les patients colligés, présentaient un tableau clinique évocateur d’un syndrome de cholestase clinico-biologique. Et le diagnostic final était établi à partir du dossier médical, radiologique et chirurgical de chaque patient. L’IRM a permis de poser le diagnostic avec une sensibilité de 88%. L’IRM avait montré chez tous les patients un obstacle biliopancréatique tumoral ou lithiasique excepté un cas où il y avait un syndrome obstructif sans obstacle biliaire ou pancréatique. Chez ce patient la chirurgie n’avait pas retrouvé d’étiologie.

## Discussion

La revue de la littérature, des obstructions des voies biliaires [2] en ce qui concerne les données cliniques et biologiques, est assez concordante à notre série. Ces données sont très importantes et d’un apport considérable pour le radiologue dans la corrélation radio-anatomique des résultats. Plusieurs étiologies ont été retrouvées: la tumeur de Klatskin ou cholangiocarcinome extrahépatique était retrouvée dans 07(41%) cas. Son pronostic spontané est sombre, car le diagnostic est fait le plus souvent à un stade évolué et survient chez un sujet âgé (+60 ans). C’est la plus fréquente (60 – 70%) des tumeurs biliaires. L’imagerie du cholangiocarcinome est complexe, mais essentielle au diagnostic compte tenu de la difficulté à obtenir une confirmation cytologique et histologique préopératoire. Elle est également indispensable pour évaluer l’extension tumorale et ainsi déterminer la résécabilité de la tumeur, dont le pronostic est lié au geste chirurgical. L’échographie est encore systématiquement effectuée devant tout tableau clinique d’ictère cholestatique. Elle confirme, et localise, environ 94 % des obstructions biliaires, en montrant des signes directs (masse hilaire, intraluminale ou d’un épaississement de la paroi biliaire) et / ou indirects (dilatation isolée des VBIH avec normalité des VBEH). Mais elle permet en même temps d’éliminer les autres causes de dilatation des VBIH. La dilatation isolée des VBIH constitue le signe le plus fréquent en imagerie. Très souvent la dilatation est bi lobaire, mais elle peut être aussi limitée à un seul lobe. La topographie et la morphologie de la dilatation biliaire sont la clé pour faire le diagnostic. Dans notre série, l’échographie a montré une dilatation des VBIH chez tous les patients porteurs de cholangiocarcinome extrahépatique. Et parmi ces patients deux avaient présenté une masse hilaire. Par contre le caractère uni ou bi lobaire n’a pas été précisé. Ceci pourrait s’expliquer du fait du caractère opérateur-dépendant de l’échographie mais aussi des limites de cette technique dans la détection de certaines formes (infiltrante). Ces résultats sont superposables à ceux de la littérature où la dilatation était retrouvée dans 85 à 100% des cas [3]. La TDM est demandée en deuxième intension en complément de l’échographie en cas de non disponibilité et ou de non accessibilité à l’IRM. Elle donne des renseignements beaucoup plus approfondis que l’échographie et à une sensibilité proche de l’IRM dans l’évaluation de l’extension vasculaire. Elle est plus performante dans la recherche de métastase à distance. Dans les formes exophytique et/ou infiltrante, leur sensibilité est proche. A l’échographie comme à la TDM, la tumeur n’est pas visualisée le plus souvent. On détecte seules les conséquences de l’infiltration. Mais le scanner comparé à l’échographie présente l’avantage de mettre en évidence un rehaussement des parois des voies biliaires au temps artériel, puis un aspect isodense au parenchyme hépatique au temps portal, persistant au temps tardif. Elle caractérise aussi la masse dans la forme exophytique, qui ne prend pas le contraste au temps artériel et se rehausse au temps portal, persistant au temps tardif. Cette cinétique est caractéristique des tumeurs de klatskin. En plus de poser le diagnostic, elle permet de faire un bilan d’opérabilité.

Dans notre série, la TDM avait permis de suspecter le diagnostic dans 56% des cas, devant la dilatation des VBIH. Elle a posé le diagnostic dans 03 cas dont 01 cas de forme infiltrante et 02 cas de masse exophytique. Ces résultats pourraient s’expliquer du fait des limites du scanner dans l’apport au diagnostic topographique de certaines formes de tumeurs des voies biliaires. Ces résultats sont conformes à ceux de la littérature qui rapporte que, le plus souvent, la tumeur de klatskin n’est pas visualisée à la TDM [4]. Mis à part, la dilatation des VBIH, qui constitue le signe indirect le plus fréquent, aucun autre signe indirect n’a été rapporté sur les observations. Il s’agit de la rétraction capsulaire et du complexe « atrophie-hypertrophie » hépatique. Les performances de la TDM sont légèrement inférieures à celles de la bili-IRM dans le diagnostic topographique des obstructions tumorales des voies biliaires [3]. Ce qui pourrait expliquer, dans notre série chez 03 cas où l’IRM était demandée d’emblée en deuxième intention. Une bili-IRM première est indiquée car il sagit du meilleur examen pour obtenir de façon non invasive une cartographie complète des voies biliaires intra et extra-hépatiques. Elle permet de localiser avec précision l'obstacle (sensibilité supérieure à 95%), de visualiser les structures canalaires exclues du fait d'une sténose, précisant ainsi l'extension locale et peut souvent identifier la nature de l’obstacle. La Bili-IRM permet de mettre en évidence les trois formes de tumeurs de Klatskin et d’établir une classification en fonction du mode d’envahissement selon Bismuth-Colrette. Sa place est prépondérante en ce qui concerne les formes infiltrantes et endoluminales dont le diagnostic est posé devant une dilatation des VBIH en amont d’une image d’arrêt abrupte. Dans notre étude, elle était demandée en troisième intention chez 14 patients et en deuxième intention chez 03 patients. Elle a mis en évidence dans 71% des cas, des lésions qui se présentaient sous forme d’image d’arrêt abrupte avec dilatation des VBIH d’amont contrastant avec un aspect normal de la VBP (aspect typique des formes infiltrant). Alors que la TDM ne retrouvait que des signes indirects. La forme exophytique était retrouvée dans 02(28%) avec un envahissement du tronc porte dans un cas. Ces résultats sont superposables à ceux de la littérature où les formes infiltrantes étaient de loin supérieures aux autres formes avec 84% contre 16% [5].

Dans notre série, le type II de Bismuth-Corlette, était retrouvé dans 70% des cas contre 14% pour le type III et IV. Des résultats similaires ont été retrouvés dans la thèse d’A. Vienne [6], où le type II prédominait. L’IRM avec des séquences cholangiographiques fines est l’examen le plus performant pour le bilan préopératoire de l’atteinte hilaire. Sa sensibilité est comparable à celle de la CPRE, utilisée comme référence, dans la série de Fulcher et al. [7] où la bili-IRM a permis une visualisation plus détaillée de la voie biliaire que la CPRE chez 3 sur 4 patients. Yeh et al. [8] ont comparé l’efficacité de la bili-IRM et de la CPRE chez 40 patients avec un obstacle malin péri-hilaire. La bili-IRM a été supérieure à la CPRE pour déterminer l’extension des lésions tumorales. Toutefois, sa place est à rediscuter face aux performances des scanners multibarettes. En effet, ceux-ci avec leurs coupes fines permettent l’obtention d’images Min-IP avec une résolution spatiale meilleure à celle de la bili-IRM. Ces scanners ont permis d’améliorer nettement les résultats quant à la détection et à la caractérisation du cholangiocarcinome. Dans la série de Tilich et al. [9], la TDM a correctement détecté tous les cholangiocarcinomes hilaires en utilisant une technique biphasique. Dans la série de C Valls [10], la TDM en plus de la détection des lésions, a permis une évaluation correcte de la classification de Bismuth-Corlette. Une des limites de la TDM est cependant l’évaluation de l’extension biliaire des petites lésions [11]. Toutefois, l’association de la TDM et la cholangio-IRM semble donc la meilleure combinaison pour le diagnostic de l’ictère et le bilan préopératoire du cholangiocarcinome hilaire.

Le cancer de la vésicule biliaire (CVB) était retrouvé dans 12% cas. L’adénocarcinome est la forme histologique la plus fréquente. C’est un cancer relativement rare qui représente 3% de toutes les tumeurs malignes et se classe au 5^ème^ rang des cancers digestifs. Le diagnostic est posé le plus souvent à un stade tardif, c'est-à-dire au stade de non résécabilité. Il survient chez des sujets âgés (60-70 ans) [3] avec une légère prédominance féminine. Son pronostic est mauvais du fait de l’envahissement précoce du foie, des ganglions du pédicule hépatique et de la rapidité de l’extension métastatique à distance [3]. L’échographie reste toujours l’examen de première intention, et suffit parfois à poser le diagnostic et à faire une première approche de l’extension de la lésion. Elle a permis dans notre étude de poser le diagnostic en montrant un épaississement tumoral focal pariéto-dépendant et une dilatation des voies biliaires, sans infiltration hépatique significative. La TDM est réalisée en complément de l’échographie et a permis de poser le diagnostic dans 60 à 74% des cas. Elle est inférieure à l’échographie pour le diagnostic d’épaississement débutant, mais elle peut être utile pour explorer une paroi vésiculaire calcifiée ou située en arrière des calculs. L’envahissement hépatique est parfois sous-estimé mais son intérêt réside essentiellement dans l’évaluation de l’extension tumorale locale et à distance. La sensibilité de ces deux techniques est proche dans le diagnostic du cancer de la vésicule biliaire dans sa forme d’épaississement focal. Une série Tunisienne a montré une sensibilité de 77 et 85% [11]. Dans notre étude, tous les patients avaient bénéficié des deux techniques qui ont permis de retenir le diagnostic dans tous les cas (100%). Cette sensibilité élevée s’explique du fait de l’étroitesse de notre échantillon mais aussi de la forme de la tumeur. Elles ont toutes montré une vésicule biliaire tumorale multi lithiasique avec dilatation des VBIH. Le scanner thoraco-abdominopelvien avec injection est actuellement la référence pour l’extension locorégionale et la recherche de métastases à distance. Sa sensibilité reste faible pour l’envahissement ganglionnaire et la carcinose péritonéale. Dans les stades avancés et devant la présence d’un ictère, une cholangio-IRM permet une meilleure étude de l’arbre biliaire et précise s’il y a ou non un envahissement hépatique par contigüité, de la voie biliaire principale (VBP), ganglionnaire ou vasculaire. La Bili-IRM était demandée en troisième intention, dans notre série, en complément de la TDM dans le bilan préopératoire. Elle avait mis en évidence en plus de la tumeur, une extension de la lésion au hile dans les deux cas et aux segments du foie (I, IV). Ces patientes étaient classées non résécables. Les performances diagnostiques de l’IRM et ses limites sont proches de celles de la TDM. La bili-IRM est plus performante pour la détection des atteintes de la voie biliaire principale et des envahissements vasculaires lorsqu’elle est associée à une angio-IRM [12]. La lithiase de la voie biliaire principale (LVBP) représentait 29% des cas. En Afrique elle est assez rare et atteint 4,8% de la population adulte jeune [2].

Le diagnostic de LVBP est facilement suspecté devant la triade classique de Villard et Perrin (douleur-fièvre-ictère) qui est observée dans 50% des cas. L’ictère survient dans la plupart des cas dans les 24 et 48 heures suivant le début de la douleur. En cas de douleur abdominale évoquant une douleur biliaire, l’échographie est l’examen de première intention. Il représente la prolongation logique de tout examen clinique. Il est facilement accessible et d’une innocuité totale. Pour le diagnostic de lithiase de la voie biliaire principale, les performances de l’échographie sont très variables d’une étude à l’autre. De 84% pour certains auteurs, elles sont de 77 à 90 % pour des opérateurs expérimentés et de 37 à 47% pour des opérateurs moins expérimentés. La sensibilité chute considérablement si les calculs sont de petite taille, s’il existe un empierrement cholédocien et si la voie biliaire n’a pas pu être explorée dans sa totalité [13]. Dans notre série, sur 05 patients présentant une lithiase du cholédoque, l’échographie avait retrouvé dans la totalité des cas des signes indirects à type de dilatation des VBIH et dans 40% des cas une LVBP. Ces résultats concordent avec les données de la littérature. Les performances de la TDM dans le diagnostic des LVBP sont nettement supérieures à celles de l’échographie, mais elle n’est demandée qu’en cas d’échec technique de l’échographie. Dans notre étude la TDM était demandée en deuxième intention chez tous les patients. Et avait permis de poser le diagnostic de LVBP. Cette technique, non invasive et facilement accessible, reste une alternative très intéressante pour le diagnostic de calcul de la VBP mais elle n’a de valeur que positive. Une série d’études comparant la TDM et l’EE avaient retrouvé une sensibilité entre 80 et 90% [14]. La plupart des études dans la littérature ont comme référence la cholangiopancréatographie rétrograde endoscopique (CPRE). Les chiffres de concordance entre ces deux examens pour le diagnostic de la LVBP sont excellents avec des chiffres de sensibilité s’échelonnant de 81% à 100% et de spécificité de 93% à 100%. Dans notre série, elle avait permis de poser le diagnostic dans tous les cas. Elle a retrouvé dans 40 des cas une lithiase unique enclavée dans le bas cholédoque et dans 60% des cas un empierrement cholédocien. Les premières études consacrées à l'évaluation de la bili-IRM ont démontré ses capacités pour le diagnostic de dilatation des voies biliaires et pour le dépistage de lithiase. La sensibilité de la bili-IRM pour le diagnostic de lithiase varie de 81 à 100 % selon les études. La bili-IRM présente des limites en ce qui concerne le diagnostic des micro-lithiases, mais a l’avantage en plus de poser le diagnostic de LVBP de mettre en évidence des variantes anatomiques des voies biliaires et d’autres pathologies associées.

Le cancer du pancréas a été retrouvé dans 02(12%) cas. Son pronostic est mauvais avec une survie globale inférieure à 3% à 5 ans. Au moment du diagnostic, 85% des tumeurs ont franchi les limites de la glande pancréatique et seules 5 à 22 % des tumeurs sont chirurgicalement résécables. Le diagnostic est le plus souvent évoqué lorsqu’il existe des symptômes spécifiques résultant de l’envahissement ou de l’obstruction de structures de voisinage. Le but principal de l’imagerie sera donc de faire un bilan d’extension de la maladie le plus précis possible afin de choisir les patients pouvant bénéficier d’un traitement curatif (résection chirurgicale) des patients relevant d’un traitement palliatif. L’échographie reste l’examen d’imagerie de première intention dans le bilan d’un ictère ou d’une douleur abdominale. Son rôle est double: établir le diagnostic positif de tumeur du pancréas et participer au bilan d’extension locorégional. Parfois, elle sera le seul examen d’imagerie nécessaire (cancer localement très avancé, métastases hépatiques). La sensibilité de l’échographie pour le diagnostic de cancer du pancréas est très variable, comprise entre 55 et 90 %. Elle dépend essentiellement de la taille et de la localisation de la tumeur. La TDM est l’examen fondamental pour le diagnostic et le bilan d’extension du cancer du pancréas [15]. Elle est l’examen à réaliser systématiquement lorsque l’échographie n’a pas retrouvé de critères formels de non résécabilité. Son but est double: affirmer le diagnostic de cancer du pancréas et effectuer le bilan d’extension locorégional et à distance le plus précis possible. Les performances de la TDM pour le diagnostic de tumeur sont excellentes dans les principales séries radiologiques avec une sensibilité dépassant le plus souvent 90 % [16]. Les principales limites (relatives) de la TDM pour le diagnostic de tumeur du pancréas sont: de faire le diagnostic positif des tumeurs de moins de 20 mm et les tumeurs isodenses sans retentissement canalaire. La place de l’IRM dans le diagnostic et le bilan d’extension des adénocarcinomes du pancréas ne sont toujours pas clairement établis. Le principal avantage de l’IRM est sa meilleure sensibilité dans le diagnostic des signes directs de tumeur du pancréas en comparaison avec l’échographie et la TDM. Ceci est particulièrement vrai pour les tumeurs de petite taille ne déformant pas les contours de la glande pancréatique. La sensibilité de l’IRM dans le diagnostic de tumeur du pancréas est très bonne, proche de 90 % et plusieurs études ayant comparé l’IRM à la TDM ont retrouvé une supériorité de l’IRM en termes de détectabilité tumorale [17]. L’échographie avait montré un processus tissulaire hypoéchogène hétérogène de la tête du pancréas et une dilatation des voies biliaires dans tous les cas. Ces résultats sont proches de la littérature (55 et 90%) et s’accordent avec les différentes études réalisées, notamment la série de Dakar qui a retrouvé une sensibilité de 89 % et une spécificité de 100% [18]. La TDM était demandée en deuxième intention systématiquement après l’échographie dans le but de rechercher des critères de résécabilité. Elle avait retrouvé en plus, de la masse pancréatique et de la dilatation des voies biliaires, une dilatation du wirsung. L’association des deux signes indirects sus-décrits réalise le classique signe de la dilatation bi-canalaire « double-duct sign » des auteurs anglo-saxons, très évocateur même lorsqu’il est isolé, du diagnostic de cancer de la tête du pancréas [19]. Une infiltration de la graisse péri-pancréatique était notée sans envahissement vasculaire. Les lésions étaient spontanément hypodenses et se rehaussaient faiblement après injection de contraste.

La sensibilité de la TDM dans cette forme est comparable à celle de l’endoscopie comme l’avait démontré une étude récente [1], comparant les performances de la TDM et de l’échographie endoscopie (EE). L’IRM, étant le gold standard dans la recherche de l’envahissement vasculaire, était demandée en troisième intention en complément de la TDM. Et avait permis de confirmer le diagnostic et l’absence d’envahissement vasculaire et ou de carcinose péritonéale. Ces résultats sont conformes à l’étude de Park HS et al [8] qui a montré qu’il n’y avait pas de supériorité statistiquement significative en ce qui concerne la sensibilité de l’IRM par rapport à la TDM dans l’extension vasculaire des tumeurs du pancréas. Ces constations prouvent que l’IRM ne doit être demandée qu’à visée diagnostique surtout pour des tumeurs de petite taille (≤ 20mm) ne déformant pas les contours pancréatiques et celles isodenses au pancréas en TDM, à visée pré thérapeutique dans le bilan d’extension hépatique, ganglionnaire et péritonéal. Certes, notre échantillon est insuffisant pour réaliser des tests statistiques significatifs, mais représentatif des principales étiologies de sténoses des voies biliaires, puisqu'elles sont toutes représentées, sauf les sténoses des anastomoses bilio-digestives, et inflammatoires qui ne faisaient pas partie de nos critères d'inclusion. Notre étude comporte un biais de recrutement lié le plus souvent à la non résécabilité de la majorité des cas des obstructions des voies biliaires. En effet tous les patients inclus dans l’étude avaient bénéficié d’un acte chirurgical. Nous avons retrouvé une bonne concordance anatomopathologique (88%) entre les résultats de bili-IRM et les données de la chirurgie. Ces résultats sont proches de ceux d’Adamek qui a été un des premiers auteurs à évaluer la valeur diagnostique de la bili-IRM pour la nature hypothétique de la sténose, montrant une sensibilité de 84% pour les lésions malignes et 100% pour les lésions bénignes. En pratique compte tenu de leur lenteur d’évolution, de leur caractère souvent infiltrant et de leur découverte tardive, les tumeurs hilaires malignes sont rarement résécables au moment du diagnostic. Ce qui concorde avec les résultats de notre étude où tous les patients étaient non résécables au moment du diagnostic. Ils ont tous bénéficié d’un traitement palliatif à type de dérivation bilio-digestive. Une bonne concordance entre les données de la chirurgie et de la Bili-IRM était observée dans la LVBP, le CVB et dans les tumeurs de la tête du pancréas. Notre bonne concordance, est probablement liée d’une part à l’étroitesse de l’échantillon mais d’autre part du fait que les obstructions des voies biliaires, surtout tumorales, s’installent à bas bruit est que leur diagnostic se fait à un stade avancé.

L’IRM dans le diagnostic des ictères cholestatiques doit être demandée en première intention chez un patient ayant des antécédents de cholécystectomie et si le risque de LVBP est réel. Dans ce contexte elle offre la meilleure imagerie pour le diagnostic de calcul résiduel. Dans les obstructions biliaires d’origine tumorale, la bili-IRM est plus performante dans la précision de l’extension biliaire, notamment en cas de tumeur infiltrante et doit être demandée en deuxième intention après l’échographie. Elle peut être demandée en troisième intention en complément de la TDM surtout dans le bilan préopératoire. C Valls [10], a montré dans sa série que l’association de la TDM et de la cholangio-IRM semble donc la meilleure combinaison pour le diagnostic et le bilan préopératoire du cholangiocarcinome avec une valeur prédictive positive (VPP) de résécabilité de 86%.

## Conclusion

La bili-IRM est l’examen de choix dans l’exploration des ictères cholestatiques. Elle doit être demandée en première intention lorsqu’on suspecte une lithiase résiduelle et en deuxième intention après l’échographie lorsque cette dernière suspecte une obstruction tumorale des voies biliaires. Son association avec la TDM constitue la meilleure combinaison pour le diagnostic étiologique et le bilan préopératoire des obstructions biliaires tumorales.

### Etat des connaissances actuelle sur le sujet

- L’ictère cholestatique est un signe d’appel d’une atteinte de l’arbre bilio-pancréatique;
- Les étiologies sont dominées par les obstructions biliaires tumorales;
- La bili-IRM est le gold standard pour l’étude de l’arbre bilio-pancréatique.

### Contribution de notre étude à la connaissance

- La bili-IRM doit être demandée en première intention en cas de suspicion d’une lithiase biliaire résiduelle;
- L’assoction bili-IRM et TDM multi détecteur constitue la meilleure combinaison pour le diagnostic étiologique et le bilan préopératoire des obstructions biliaires tumorales.

## References

[1] Dupas B, Gournay J, Frampas E, Leaute F, Le Borgne J (2006). Une cholestase anicterique: l’apport de l’imagerie au diagnostic étiologique. J Radiol.

[2] Kondo S, Isayama H, Akahane M, Toda N, Sasahira N, Nakai Y (2005). Detection of common bile duct stones: comparison between endoscopic ultrasonography, magnetic resonance cholangiography and helical-computed-tomographic cholangiography. Eur J Radiol.

[3] Hammel P (2006). Cancer du pancréas: quoi de neuf en 2005?. Bull Cancer (Paris).

[4] Vilgrain V, Paradis V (2010). Cholangiocarcinome périphérique: corrélations radiopathologiques. Gastroentérologie Clin Biol.

[5] Taourel P, Calvet C, Lecesne R, Pradel JA, Drouillard J, Bruel JM (1997). Apport de la cholangiopancréatographie IRM dans la pathologie des voies biliaires et pancréatiques. J Radiol.

[6] Vienne A, Chryssostalis A, Hobeika E, Lapidus N, Ducot B, Fritsch J (2008). Etude des facteurs prédictifs de succès du drainage endoscopique des sténoses hilaires tumorales. Endoscopy.

[7] Fulcher AS, Turner MA (1997). HASTE MR cholangiography in the evaluation of hilar cholangiocarcinoma. Am J Roentgenol.

[8] Yeh TS, Jan YY, Tseng JH, Chiu CT, Chen TC, Hwang TL (2000). Malignant perihilar biliary obstruction: magnetic resonance cholangiopancreatographic findings. Am J Gastroenterol.

[9] Tillich M, Mischinger HJ, Preisegger KH, Rabl H, Szolar DH (1998). Multiphasic helical CT in diagnosis and staging of hilar cholangiocarcinoma. Am J Roentgenol.

[10] Valls C (2006). L’ictère nu: rôle du radiologue dans la prise en charge diagnostique et thérapeutique. J Radiol.

[11] Belkheir M (1997). Le cancer de la vésicule biliaire. Thèse de médecine.

[12] Issouami R (2012). AmpullomeVatérien.

[13] Masson E Cancers de la vésicule biliaire. Technique chirurgicale [Internet].

[14] O’Toole D, Palazzo L (2006). Choledocholithiasis - a practical approach from the endosonographer. Endoscopy.

[15] Takhar AS, Palaniappan P, Dhingsa R, Lobo DN (2004). Recent developments in diagnosis of pancreatic cancer. BMJ.

[16] Levy P, Collectif d’auteurs (2009). hépato-gastro-entérologie. Issy-les-Moulineaux: Elsevier Masson SAS Coll. Abrégés - Connaissances et pratique.

[17] Venniyoor A (2008). Cholesterol gallstones and cancer of gallbladder (CAGB): molecular links. Med Hypotheses.

[18] Frouge C (1996). La cholangiographie par résonance magnétique. J Radiol.

[19] Park HS, Lee JM, Choi HK, Hong SH, Han JK, Choi BI (2009). Preoperative evaluation of pancreatic cancer: comparison of gadolinium-enhanced dynamic MRI with MR Cholangiopancreatography versus MDCT. J Magn Reson Imaging.

